# Dengue fever in Bangladesh: rising trends, contributing factors, and public health implications

**DOI:** 10.1186/s40794-025-00251-6

**Published:** 2025-08-11

**Authors:** Ikponmwosa Jude Ogieuhi, Mohamed Mustaf Ahmed, Safayet Jamil, Olalekan John Okesanya, Bonaventure Michael Ukoaka, Gilbert Eshun, Jerico Bautista Ogaya, Don Eliseo Lucero-Prisno III

**Affiliations:** 1 Siberian State Medical University, Tomsk, Russia; 2 Faculty of Medicine and Health Sciences, SIMAD University, Mogadishu, Somalia; 3 Department of Public Health, Daffodil International University, Dhaka, 1216 Bangladesh; 4 Department of Public and Community Health, Faculty of Medicine and Health Sciences, Frontier University, Garowe, Puntland, Somalia; 5 Department of Public Health and Maritime Transport, University of Thessaly, Volos, Greece; 6 Department of Medical Laboratory Science, Neuropsychiatric Hospital, Aro, Abeokuta, Federal Republic of Nigeria; 7 Department of Medical Laboratory Science, Chrisland University, Ajebo, Abeokuta, Nigeria; 8 Community and Clinical Research Division, First On-Call Initiative, Port Harcourt, Nigeria; 9 The Royal (Dick) School of Veterinary Studies, University of Edinburgh, Edinburgh, UK; 10 Department of Medical Technology, Institute of Health Sciences and Nursing, Far Eastern University, Manila, Philippines; 11 Center for University Research, University of Makati, Makati City, Philippines; 12 Department of Global Health and Development, London School of Hygiene and Tropical Medicine, London, UK; 13 Center for Research and Development, Cebu Normal University, Cebu, Philippines

**Keywords:** Dengue fever, Epidemiology, Bangladesh, Good health and well-being, Public health

## Abstract

Dengue fever has emerged as a major public health crisis in Bangladesh, with an unprecedented surge in cases and fatalities in recent years. This paper analyzed the epidemiological trends, contributing factors, and public health implications of the rising dengue burden in the country. Surveillance data revealed a staggering 203,406 dengue cases and 989 deaths between January and September 2023, marking a 1.9-fold increase compared with the entire year of 2019. The capital bears the brunt of the outbreak and accounts for over half of all cases and deaths. Climatic factors, rapid urbanization, population density, insecticide resistance, and a lack of public awareness have created a perfect storm for dengue transmission. The overburdened healthcare system struggles to cope with the influx of patients, leading to a compromised quality of care and economic strain. Vulnerable populations have a heightened risk of developing severe complications and mortality. This paper highlights the urgent need for a multipronged approach encompassing surveillance, case management, vector control, risk communication, and community engagement to combat the dengue epidemic in Bangladesh. Sustained political commitment, adequate resources, and strong multi-sectoral collaboration are imperative to reduce the disease burden and safeguard public health in the face of this escalating threat.

## Introduction

Dengue fever (DF), a mosquito-borne viral disease, has emerged as a significant public health concern in Bangladesh. The disease is caused by the dengue virus, which belongs to the Flaviviridae family and has four distinct serotypes (DENV-1, DENV-2, DENV-3, and DENV-4) [1]. Transmission occurs through the bite of infected female Aedes mosquitoes, primarily *Aedes aegypti*. The clinical manifestations of dengue fever vary widely, ranging from mild flu-like symptoms to severe and potentially life-threatening conditions such as dengue hemorrhagic fever (DHF) and dengue shock syndrome (DSS). Typical symptoms include high fever, severe headache, retro-orbital pain, myalgia, arthralgia, and characteristic skin rash. In some cases, the disease can progress to severe dengue, characterized by plasma leakage, severe bleeding, and organ involvement [2, 3]. Despite the absence of specific treatments for dengue, timely identification and appropriate case management are crucial to reduce fatality rates [4]. Bangladesh has been experiencing an increasing trend in dengue cases over the past few decades. The first recorded outbreak of dengue in the country, known as East Pakistan, occurred in the 1960s and was referred to as “Dacca fever” [5]. Since 2000, dengue outbreaks have become more frequent and widespread, with a notable surge in cases occurring during the monsoon season, from May to September [6]. This heightened transmission period is attributed to factors such as excessive waterlogging, flooding, heavy rainfall, and rising temperatures, which are conducive to the spread of dengue and other mosquito-borne diseases, such as malaria and chikungunya.

### Epidemiological trends

**Fig. 1:**
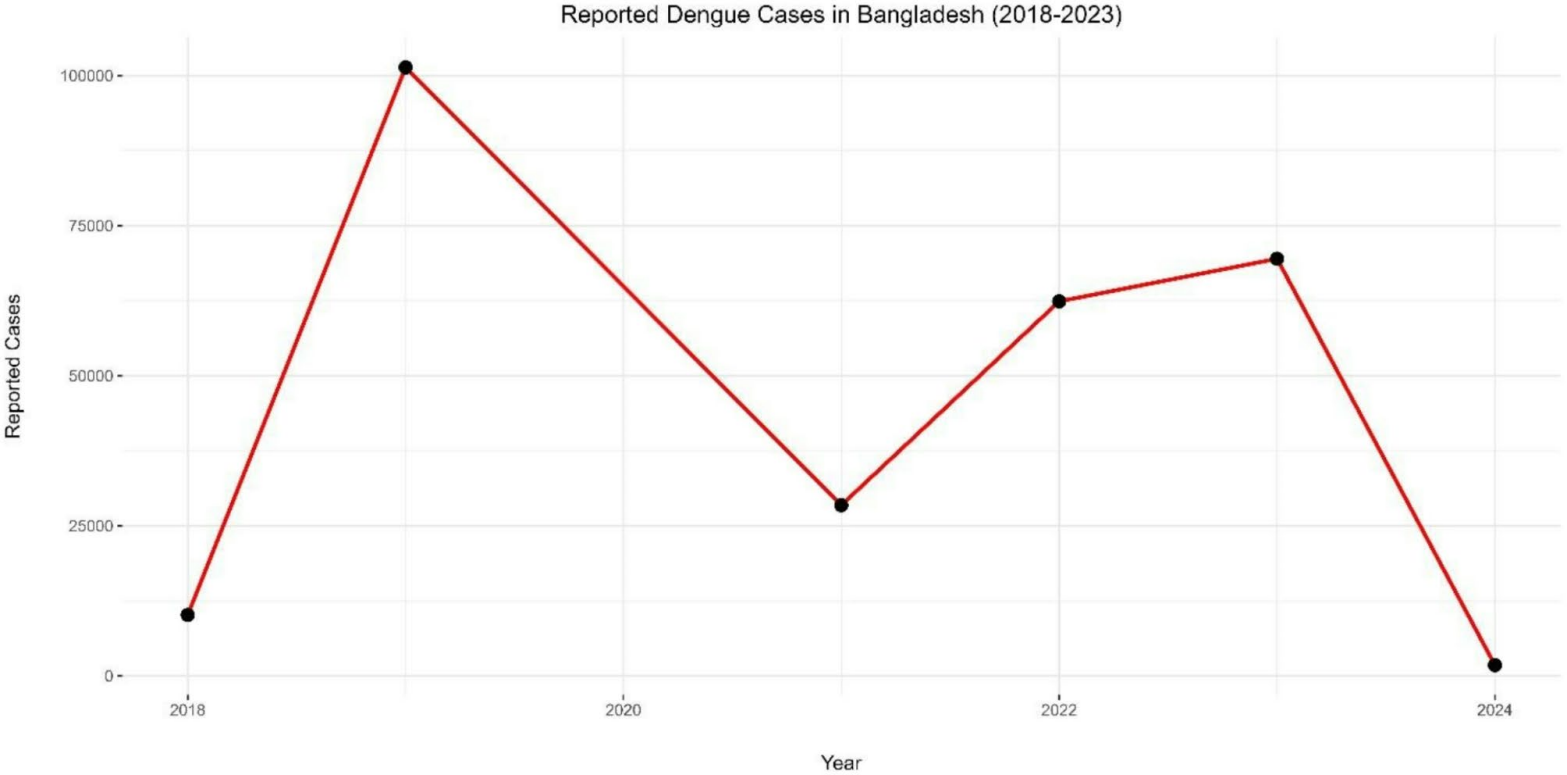
Annual reported dengue cases in Bangladesh (2018–2023)

**Fig. 2:**
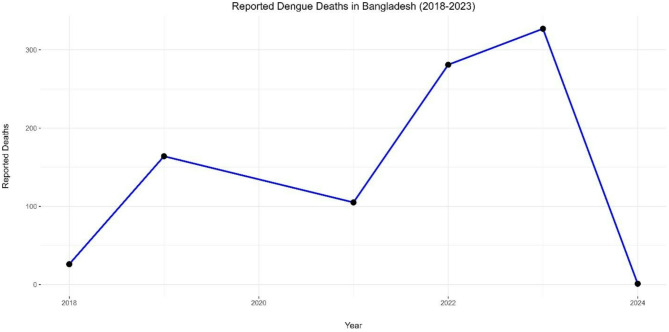
Annual reported dengue deaths in Bangladesh (2018–2023)

The dengue outbreak in Bangladesh has taken a dire turn in 2023, with an unprecedented number of reported cases and deaths (Figs. 1 and 2). According to the Directorate General of Health Services (DGHS), a cumulative total of 203,406 dengue cases and 989 deaths were recorded between January 1 and September 30, 2023 [7]. This represents a significant increase compared to the incidence recorded during the same period in the previous year. As of June 30, 2023, 7,978 cases and 47 deaths were reported. However, a dramatic surge in cases began in late June, with 204 deaths recorded in July alone [8]. The number of cases continued to increase, surpassing the highest point observed since 2000. Notably, within just three months from July to September, the recorded cases were 1.9 times the total cases reported in the entire year of 2019, which was the last major outbreak in Bangladesh when the country experienced its worst outbreak, with 101,354 reported cases and 179 deaths [4].

All 64 districts in Bangladesh have reported dengue cases, with Dhaka being the most affected area, accounting for 52.8% of cases and 78.9% of deaths. Other significantly impacted divisions included Chattogram (13.2% of cases and 9.2% of deaths), Barisal (10.5% of cases and 4.3% of deaths), and Sylhet, which recorded the lowest number of cases (560) and no deaths thus far [9]. The Rohingya refugee camps in Cox’s Bazar have also emerged as significant hotspots for dengue transmission in Banglades [10]. Dengue fever is a serious health concern in the Rohingya refugee camps in Cox’s Bazar, Bangladesh. Camps are vulnerable to infectious disease outbreaks due to factors such as overcrowding and inadequate access to healthcare [11]. The current dengue situation (as of June 5, 2024) in the camps showed a 56% decrease in reported cases compared to the previous week, with 28 cases reported. The cumulative number of dengue cases in 2024 is 1,792, with one death resulting in a 0.1% fatality rate (Table 1). Approximately 16% of dengue cases in 2024 are moderate to severe, requiring hospital admission [12]. Surveillance and control efforts in these camps are crucial to manage the overall dengue situation in Bangladesh.

**Table 1:** Dengue cases and deaths in Bangladesh: annual trends (2018–2023)*

Year	Reported Cases	Reported Deaths	Case Fatality Rate (CFR)
2018	10,148	26	0.26%
2019	101,354	164	0.16%
2021	28,429	105	0.37%
2022	62,382	281	0.45%
2023	69,483	327	0.47%
2024	1,792	1	0.1%

*Data source: DGHS (Data for 2020 is limited due to COVID-19)

### Factors contributing to rising dengue cases

The increasing prevalence of dengue fever in Bangladesh can be attributed to several interconnected factors that can be broadly categorized into environmental, demographic, and socioeconomic themes.

### Environmental factors

Climate is one of the major factors known to influence the generation of mosquitoes and the transmission of the dengue virus (Table 2). A study conducted in Dhaka City, Bangladesh, found a substantial correlation between monthly reported dengue cases and meteorological parameters, such as rainfall, maximum temperature, and relative humidity [13]. These findings suggest that climate change has a major effect on the occurrence of dengue in cities [14]. Numerous studies have identified the significance of climatic factors in dengue transmission. Temperature plays a crucial role in the rapid replication of dengue virus within the mosquito vector, whereas humidity increases vector survival and transmission potential [13]. High temperatures can shorten the extrinsic incubation period of viruses in mosquitoes, leading to more frequent transmission cycles. Precipitation also affects mosquito egg availability and larval breeding sites, as stagnant water from heavy rain provides ideal conditions for mosquito breeding. The spread and occurrence of dengue fever are worsening owing to climate change, which elevates temperatures and alters precipitation patterns, broadens mosquito habitats, and quickens the lifecycle of mosquitoes [15]. Projections indicate that Bangladesh is likely to experience a longer dengue fever season in the future, owing to climate change [16].

### Urbanization and population density

Rapid urbanization and increased population density are also key factors contributing to the increasing number of dengue cases in Bangladesh. Urbanization frequently results in overcrowding, poor infrastructure, and unhealthy living conditions. These factors contribute to the creation of optimal breeding grounds for Aedes mosquitoes, which are primary vectors for dengue virus transmission [17]. In urban areas, water storage practices, such as storing water in containers owing to irregular water supply, can provide breeding sites for mosquitoes. Poor sanitation practices, such as the inappropriate disposal of solid waste and inadequate drainage systems, provide breeding sites for mosquitoes in stagnant waters, further increasing the risk of dengue transmission [18]. Moreover, a high population density increases the probability of contact between mosquitoes and humans, facilitating the spread of the virus.

### Public awareness and preventive measures

The lack of public awareness regarding dengue prevention and symptoms among the population is another factor implicated in the increased incidence of dengue [19]. Many people are unaware of how to prevent mosquito bites or eliminate breeding sites, leading to a higher exposure to infected mosquitoes. Additionally, Aedes mosquitoes have developed resistance to commonly used insecticides. A study conducted in Bangladesh found that *Aedes aegypti* is resistant to permethrin, but not to deltamethrin [20, 21]. Increased enzyme levels of esterases and mixed-function oxidases (MFOs) and the presence of the kdr mutations V1016G and F1534C have been reported [22]. Insecticide resistance hampers effective vector control measures, making it challenging to reduce mosquito populations and interrupt dengue transmission.

### Vaccine availability

The availability and efficacy of dengue vaccines remain a subject of debate and have contributed to the increasing number of cases. Although WHO recommends that countries consider vaccination as part of their integrated dengue prevention and control strategy [23], routine vaccinations are not readily available, especially in remote regions and areas with high population densities in Bangladesh [24]. The limited availability of vaccines, coupled with logistical challenges in their distribution and administration, means that a significant portion of the population remains unprotected against dengue. Furthermore, vaccine efficacy can vary depending on the recipient’s previous exposure to different dengue serotypes, which complicates the implementation of a widespread vaccination program.

**Table 2:** Factors contributing to rising dengue cases

Theme	Factor	Description
Environmental	Climate Change	Climate change has a major effect on the occurrence of dengue cases, with substantial correlation between dengue cases and meteorological parameters like rainfall, temperature, and humidity. High temperatures shorten the virus’s incubation period, and precipitation provides breeding sites.
	Temperature and Humidity	Temperature plays a crucial role in the rapid replication of the dengue virus, while humidity increases vector survival and transmission potential. High temperatures can shorten the extrinsic incubation period of the virus in the mosquito.
	Precipitation	Stagnant water from heavy rains provides ideal conditions for mosquito breeding, worsening the spread and occurrence of dengue fever. Projections indicate a longer dengue fever season in the future owing to climate change.
Demographic	Urbanization and Population Density	Rapid urbanization leads to overcrowding, poor infrastructure, and unhealthy living conditions, creating optimal breeding grounds for Aedes mosquitoes. Water storage practices and poor sanitation also contribute to increased mosquito breeding.
	Water Storage Practices	Storing water in containers due to irregular water supply provides breeding sites for mosquitoes.
	Poor Sanitation Practices	Inappropriate disposal of solid waste and inadequate drainage systems provide breeding sites for mosquitoes in stagnant waters, increasing the risk of dengue transmission.
Socio-economic	Public Awareness and Preventive Measures	Lack of public awareness regarding dengue prevention and symptoms leads to higher exposure to infected mosquitoes. Resistance to commonly used insecticides hampers effective mosquito control measures.
Healthcare System	Insecticide Resistance	Aedes mosquitoes have developed resistance to commonly used insecticides, making it challenging to reduce mosquito populations and interrupt dengue transmission
	Vaccine Availability	Limited availability and logistical challenges in vaccine distribution leave a significant portion of the population unprotected. Vaccine efficacy can vary depending on previous exposure to different dengue serotypes.

### Public health implications of rising dengue

Rising dengue cases in Bangladesh have significant public health implications, placing a substantial burden on the healthcare system. The increasing number of dengue patients has led to a surge in hospitalizations, outpatient visits, and the demand for medical resources [25]. A study conducted in South India found that the median cost of hospitalization for fever, including dengue, was 4,243 Indian rupees (INR) per episode, with dengue having the highest median cost at 5,627 INR [26]. In Bangladesh, the median hospitalization cost for dengue fever can vary widely depending on the severity of the disease and the healthcare facility. However, a study conducted in Dhaka reported that the average cost to society for a dengue fever episode in urban Dhaka in 2019 was US$479.02. This cost includes expenses borne by households and healthcare providers. Households bear a substantial portion of this cost, accounting for 85% of the total cost [27]. This financial burden can be particularly challenging for low-income households.

The strain on healthcare infrastructure is evident during outbreaks, with hospitals struggling to accommodate the influx of patients. In 2023, six hospitals previously used for COVID-19 management in Dhaka City were repurposed for dengue cases to cope with increased demand [9]. An overwhelming number of patients can lead to longer wait times and a compromised quality of care. The economic impact of dengue extends beyond the direct medical costs. One study estimated that the economic burden of dengue in Southeast Asian countries reached millions of US dollars annually [28]. The indirect costs associated with lost productivity due to illness and premature death further contribute to the economic toll. A recent analysis revealed that the economic and disease burden of dengue fever surpassed that of 17 other disorders, including Japanese encephalitis, hepatitis B, and upper respiratory tract infections [28].

Vulnerable populations, such as children, pregnant women, the elderly, and those with comorbidities, face a higher risk of severe dengue complications including hemorrhagic fever and shock syndrome [29, 30]. These complications can lead to increased morbidity and mortality. A study conducted at the Rangpur Community Medical College and Hospital in Bangladesh found that, among dengue patients, 86.54% had dengue fever, 7.69% had grade 1 dengue hemorrhagic fever, and 5.77% had dengue shock syndrome [31]. The psychological impact of dengue outbreaks on the affected communities should not be overlooked. The fear and anxiety associated with the disease, coupled with disruption of daily life and potential loss of loved ones, can have significant mental health consequences [32]. A study in coastal areas of Bangladesh found that natural disasters triggered by climate change, such as cyclones, could affect the physical and mental health of the population, with children and older adults being the most vulnerable groups [33]. Due to their compromised immune systems and previous medical disorders, these vulnerable individuals are also more likely to experience severe dengue complications [34]. These public health implications of increasing dengue cases in Bangladesh highlight the urgent need for effective prevention and control measures.

Rising dengue cases also have implications for public health policy and resource allocation. The government may need to divert resources from other health programs to address the dengue epidemic, potentially affecting the management of other diseases [35]. Additionally, the increased focus on dengue may strain public health education and awareness campaigns, making it challenging to effectively address other health concerns. Furthermore, the dengue epidemic could have long-term consequences on Bangladesh’s healthcare workforce [36]. Increased workload and stress on healthcare professionals may lead to burnout and potentially impact the quality of care provided for both dengue and other health conditions. This situation underscores the need for strategies to support and expand the healthcare workforce to meet the growing demands of dengue management, while maintaining overall healthcare quality.

### Recommendation for public health intervention

Given the increasing number of dengue cases in Bangladesh, there is an urgent need for effective public health interventions to prevent and control its spread. A multipronged approach that includes surveillance, case management, vector control, risk communication, and community engagement is essential. Establishing a robust hospital-based surveillance system is key to actively collecting information from hospitals and disseminating it through emergency centers. This system should be integrated with the existing national surveillance networks to ensure timely reporting, data analysis, and response coordination [37]. Effective case management is also critical, necessitating that healthcare facilities across the country be equipped with adequate medical supplies, such as intravenous saline and supportive medicines, to effectively manage patients with dengue [38].

Vector control remains the cornerstone of dengue prevention and control. The government should prioritize the implementation of the “4S” strategy, which includes searching and destroying mosquito breeding sites, self-protection measures, seeking early medical attention for fever lasting more than 48 h, and saying yes to fogging during outbreaks [39]. Entomological surveillance should be undertaken to assess the breeding potential of Aedes mosquitoes in containers and to monitor insecticide resistance [40]. Regular larvicidal and adult mosquito control using different insecticides such as deltamethrin and temephos should be performed by both the general public and officials [41]. Risk communication and community engagement are essential components of successful dengue prevention and control programs. Proper risk communication should be strengthened through various channels, such as television, leaflets, and posters to increase awareness of dengue symptoms, prevention measures, and the importance of seeking early medical attention [42]. Community engagement should be promoted through regular advocacy and awareness meetings to encourage active participation of community members in vector control activities [43].

Personal protective measures should also be encouraged, particularly during outdoor activities. These include the use of topical repellents on exposed skin, wearing long pants and sleeves, and treating clothing with insecticides [44, 45]. Indoor protection measures such as the use of mosquito coils, nets, and air conditioning can also reduce the entry of mosquitoes into homes. It is crucial to strengthen the capacity of the healthcare system to manage dengue outbreaks. This includes training healthcare workers in dengue management, ensuring adequate supply of diagnostic tests and treatment materials, and developing protocols for patient triage and management during peak seasons. Additionally, establishing dedicated dengue treatment units in hospitals could improve patient care and reduce strain on general medical facilities. Inter-sectoral collaboration is vital for effective dengue control. Partnerships between health, urban planning, water and sanitation, and education sectors can address the root causes of dengue transmission, such as improper waste management and inadequate water supply systems. Engaging with community leaders, religious organizations, and local NGOs can enhance the reach and effectiveness of dengue prevention programmes [46]. Thus, long-term strategies should focus on research and innovation. Investing in the development of new vector control tools, improved diagnostics, and potential vaccines is crucial. Collaboration between international research institutions and participation in clinical trials could accelerate the availability of new interventions for dengue control in Bangladesh. Finally, it is essential to integrate dengue control efforts into broader climate change adaptation strategies. As climate change is likely to exacerbate dengue transmission, incorporating dengue prevention into climate resilience plans could ensure a more comprehensive and sustainable approach to public health. It is also essential to address potential challenges and barriers [47]. This may include inadequate resources, limited technical capacity, and low community participation. Strategies to overcome these challenges should be developed, such as mobilizing resources from various sources, providing regular training to health workers and community volunteers, and implementing innovative approaches to engaging communities [48].

## Conclusion

Rising dengue cases in Bangladesh present a complex public health challenge that demands urgent attention and concerted efforts from all stakeholders. The interplay of factors such as climate change, rapid urbanization, insecticide resistance, lack of public awareness, and limited access to effective vaccines creates a favorable environment for the transmission and spread of dengue. Public health implications are significant, placing a substantial burden on healthcare systems and leading to increased hospitalizations, economic impacts, and disproportionate effects on vulnerable populations. To effectively address this threat, Bangladesh must adopt a comprehensive and integrated approach that prioritizes surveillance, case management, vector control, risk communication, and community engagement. Sustained political commitment, adequate financial resources, and strong collaboration between different sectors are crucial for reducing disease burden and protecting public health. By implementing evidence-based interventions, investing in research and innovation, and empowering communities, Bangladesh can make significant strides in combating dengue epidemics. Rising dengue cases serve as a clarification call for action, urging policymakers, healthcare providers, and the public to unite in the fight against this debilitating disease. Using a holistic and proactive approach, Bangladesh can safeguard the health and well-being of its population and pave the way for a dengue-free future.

## Data Availability

No datasets were generated or analysed during the current study.
